# Delayed Irreversible Fanconi Syndrome Associated With Vertebral Fracture After Tenofovir Discontinuation

**DOI:** 10.7759/cureus.53280

**Published:** 2024-01-31

**Authors:** Ghofran N Qorban, Jameelah Alyami, Shaza Samargandy, Tariq A Madani

**Affiliations:** 1 Endocrinology, Diabetes, and Metabolism, Dr. Solaiman Fakeeh Hospital, Jeddah, SAU; 2 Endocrinology, Diabetes, and Metabolism, King Abdulaziz University Hospital, Jeddah, SAU; 3 Diabetes and Endocrinology, Dr. Solaiman Fakeeh Hospital, Jeddah, SAU; 4 Endocrinology, King Abdulaziz University Hospital, Jeddah, SAU; 5 Medicine, King Abdulaziz University Hospital, Jeddah, SAU

**Keywords:** osteoporotic fractures, bone mineral density (bmd), tenofovir alafenamide (taf), tenofovir disoproxil fumarate (tdf), human immunodeficiency virus (hiv)

## Abstract

The use of tenofovir disoproxil fumarate (TDF) as an antiretroviral agent has been reported to adversely affect both renal tubules and bone health, leading to pathological fractures. While such an effect is largely reversible, substituting TDF with tenofovir alafenamide (TAF) might result in lower rates of adverse events with the preservation of tenofovir effectiveness. We report a case of a 40-year-old lady with HIV infection who had a vertebral fragility fracture secondary to TDF-associated Fanconi syndrome. The syndrome developed four years after TDF cessation and switching to TAF. Other etiologies for decreased bone mass were excluded, and the diagnosis of Fanconi syndrome was established based on her bone mineral density (BMD) and urine parameters. She was treated conservatively with active vitamin D, calcium, and progesterone/estrogen combination, but her phosphate wasting persisted despite switching to TAF; this likely represents a delayed irreversible effect of TDF on the patient’s bone remodeling. This case report highlights the chronic sequelae of TDF therapy and the importance of monitoring for and early detection of renal tubulopathy and osteoporotic fractures in this patient population.

## Introduction

The innovation of highly active antiretroviral therapy (HAART) for patients diagnosed with HIV has led to substantial disease control and a decrease in the burden of HIV-related opportunistic infections (OIs) [1, 2]. Tenofovir disoproxil fumarate (TDF) is one of the nucleotide/nucleoside reverse transcriptase inhibitors (NRTIs) drug classes that is considered the backbone of most HAART regimens [3]. Tenofovir alafenamide (TAF) is a more recent prodrug formulated to overcome TDF nephrotoxic and skeletal adverse events while preserving class effectiveness regarding viral suppression and immune reconstitution in both drug-naïve and drug-exposed patients. Three recent systematic reviews and meta-analyses of real-world randomized controlled trials showed that TAF does carry a lower risk of adverse effects on renal and bone density parameters compared to TDF [4-6]. Additionally, switching to TAF in patients who experienced proximal renal tubulopathy while on TDF has improved renal and bone effects without the risk of recurrence of tubulopathy in susceptible individuals [7, 8]. From a pharmacologic point of view, the disoproxil fumarate substitution with alafenamide in TAF makes tenofovir more bound to it, which results in lower plasma pharmacokinetic distribution, a higher rate of clearance, and subsequently a lower toxic effect on renal tubules and bone turnover [9]. As a result, there has been a greater trend to prescribe TAF over the past few years [10].

Fanconi syndrome is a disorder that includes proximal renal tubular acidosis, proteinuria, hypokalemia, renal phosphate wasting, aminoaciduria, and hypouricemia, along with renal impairment [11]. The derangement of bone mineral density (BMD) without the formal establishment of Fanconi syndrome has been described as one of the uncommon and detrimental adverse events of TDF therapy that is reported in approximately 500 cases, which constituted almost one-third of the patients with HIV infection who received TDF as a part of their HAART regimen, according to the US Food and Drug Administration's (FDA) Adverse Event Reporting System (FAERS) [12].

In real-world data, Fanconi syndrome incidence has ranged between one and five per 1,000 patients receiving TDF [13, 14]. Although more than 50% of Fanconi syndrome cases are resolved within the first year following TDF discontinuation [15, 16], delayed recovery has been observed in some cases [17]. Herein, we report a case of TDF-associated Fanconi syndrome, which resulted in bone mass loss and fragility fracture approximately four years following TDF discontinuation.

## Case presentation

**Figure 1:**
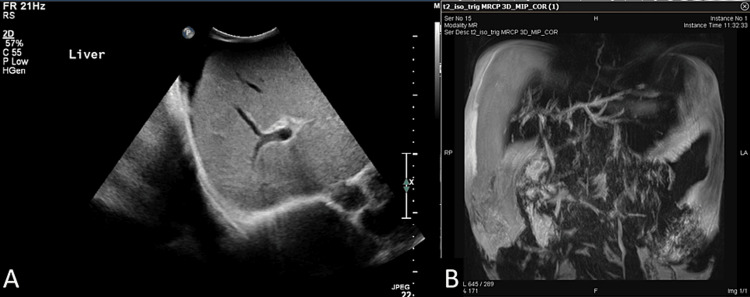
Ultrasound of the abdomen and pelvis and magnetic resonance cholangiopancreatography (MRCP) of the reported patient Figure 1A: An ultrasound of the abdomen and pelvis reveals an enlarged liver measuring 18 cm and homogeneous parenchyma with no focal lesions or intrahepatic biliary dilatation. The portal vein and common bile duct are unremarkable. The gallbladder is surgically removed. The pancreas and both kidneys are unremarkable. The spleen is enlarged, measuring 18 cm, with no focal lesions. There is no ascites. Figure 1B: The MRCP shows two focal areas of cholangitis in the right liver lobe associated with a beaded appearance, suggestive of sclerosing cholangitis with multiple unchanged liver lesions, which represents mostly hepatic abscesses; however, cystic neoplasm could not be totally excluded. There are no radiological signs of pancreatitis.

A 40-year-old female who was diagnosed with HIV infection in 2010 and lost follow-up for almost five years presented to our hospital on March 31, 2015, with acquired immunodeficiency virus syndrome (AIDS), which defines illnesses including wasting syndrome. Her vitals were as follows: weight: 25 kg, height: 155 cm, body mass index (BMI): 10.4 kg/m^2^), *Pneumocystis jirovecii* pneumonia (PCP) (hypoxemia and bilateral interstitial pulmonary infiltrates), chronic diarrhea secondary to cryptosporidiosis, severe oral candidiasis and chronic herpetic oral ulcers, pancytopenia, and cholestatic jaundice (total bilirubin: 84 umol/L, direct bilirubin: 72 umol/L, alkaline phosphatase (ALP): 1400 U/L, gamma-glutamyl transferase (GGT): 581 U/L, aspartate transferase (AST): 149 U/L, alanine transferase (ALT): 82 U/L, and renal glucose urea: 2.3 mmol/L). Abdominal ultrasound showed biliary sludge with no intrahepatic or extrahepatic biliary dilation, while magnetic resonance cholangiopancreatography (MRCP) showed an enlarged (21 cm diameter) fatty liver with mild dilatation of the intra- and extrahepatic biliary systems and multiple areas of narrowing and stricture formation with no obvious intrahepatic stone formation (Figure 1).

A liver biopsy showed chronic inflammation, a single granuloma with mild fibrosis, marked steatosis, and no evidence of malignancy. Cholangiopathy secondary to HIV or cryptosporidium with concomitant hepatic typical (tuberculosis) or atypical (*Mycobacterium avium-intracellulare* infection (MAI)) infection was diagnosed. The laboratory investigations revealed an HIV viral load of 2,566,506 copies/ml and a CD4 count nadir of 12 cells/mm3. Moreover, genotypic resistance testing revealed no significant resistance-conferring mutations. The patient was treated for her presumed hepatic tuberculosis or MAI infection with first-line anti-tuberculosis therapy (isoniazid, rifampin, ethambutol, and pyrazinamide) along with clarithromycin for MAI. She also received full treatment for PCP with cotrimoxazole and prednisone for 21 days, followed by secondary prophylaxis with cotrimoxazole, and was commenced on TDF/emtricitabine (co-formulated as Truvada®) and efavirenz on April 5, 2015. Twenty-one days after the initiation of TDF, the previously normal serum potassium (4.7 mmol/L) and phosphate (0.81 mmol/L) levels were noticed to be low (potassium: 2.8 mmol/L, phosphate: 0.36 mmol/L) with a normal magnesium level (1.11 mmol/L). Since the patient had severe anemia (hemoglobin: 5.9 g/dL) that precluded the use of zidovudine as an alternative to TDF and the unavailability of abacavir, the TDF was continued as the benefit of treating the patient’s advanced HIV infection outweighed the adverse effect of TDF-induced tubulopathy. However, when the patient’s hemoglobin improved to 9.4 g/dL, TDF/emtricitabine (Truvada®) was changed to zidovudine/lamivudine on February 10, 2016, because of persistent hypokalemia.

In April 2016, zidovudine/lamivudine was changed back to TDF/emtricitabine because of worsening anemia induced by zidovudine (hemoglobin dropped from 9.4 to 6.9 g/dL). However, in May 2016 (13 months after initiation of antiretroviral therapy (ART)), the patient’s investigations were remarkable for persistent hypokalemia (2.6 mmol/L) and hypophosphatemia (0.66 mmol/L), undetectable HIV viral load, a CD4 count of 292 cells/uL, and hemoglobin of 9.7 g/dL. Due to concerns about TDF-induced tubulopathy and the improvement of her hemoglobin level, her antiretroviral regimen was changed to zidovudine, lamivudine, and efavirenz. However, this regimen was stopped in December 2016 due to acute pancreatitis (amylase: 2620 U/L (N: 25-115) and lipase: 8015 u/L (N: 73-393)) thought to be caused by at least one of its components (TDF, emtricitabine, or efavirenz). A month later, her HIV viral load rose to 4,282,432 copies/mL, and her CD4 count dropped to 85 cells/uL. The HAART regimen was planned to be resumed after recovery from acute pancreatitis, but the patient was lost to follow-up for almost a year.

**Figure 2:**
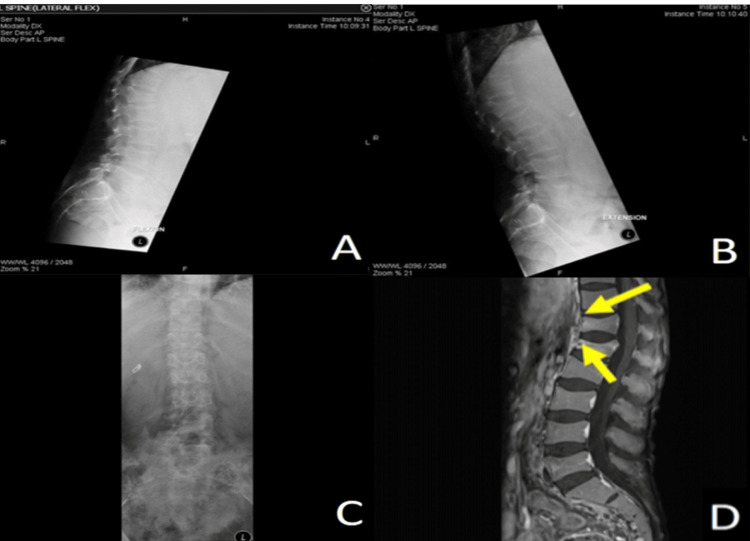
Radiological studies of the reported patient Figures 2A-2C: Lumbar spine x-ray revealing osteopenia with narrowed intra-vertebral spaces (A: lumbar spine lateral x-ray with flexion, B: lumbar spine lateral x-ray with extension, and C: lumbar spine anteroposterior x-ray). Figure 2D: The MRI confirmed a 12th-thoracic vertebral-first-lumbar vertebra (T12-L1) compression fracture with biconcave deformity of their endplates, illustrating the classical codfish radiological sign appearance (yellow arrows).

In June 2018, the patient presented again for follow-up. Her HIV viral load was 2,473,928 copies/mL, and her CD4 count was 83 cells/uL. Repeated genotypic resistance testing revealed no resistance to any nucleotide/nucleoside or non-nucleoside reverse transcriptase inhibitors or protease inhibitors. As a result, she was commenced on elvitegravir 150 mg, cobicistat 150 mg, emtricitabine 200 mg, and tenofovir alafenamide 10 mg co-formulated tables (Genvoya). However, when seen in a follow-up 14 months later, in August 2019, the patient’s viral load was not completely suppressed (1,840 copies/mL), and the CD4 count was 169 cells/uL. The patient admitted to being non-adherent to therapy. At that time, the patient complained of a dull ache in the back, generalized bone pain, and myalgia. On examination, she was cachectic (weight: 36 kg, height: 155 cm, BMI: 15 kg/m^2^), had a waddling gait with tender points at the thoracolumbar region of the spine, and had severe proximal myopathy. An MRI of the spine confirmed the presence of compression fractures of the 12th-thoracic and first-lumbar vertebrae (T12-L1) and her bone densitometry revealed a Z-score of -6.4 at the lumbar spine and -5 at the femoral neck (Figure 2).

She was subsequently investigated for the causes of low bone mass. There was evidence of low vitamin D levels, which can be attributed to a lack of sun exposure along with impaired hepatic function and the expected dysfunctional hydroxylation of vitamin D at position 25. In addition, there was a chronic state of phosphate wasting that was not treated even after stopping TDF and switching to TAF. Her laboratory work-up excluded other causes of low bone mass at a young age, such as primary hyperparathyroidism, hypogonadism, thyrotoxicosis, renal failure, and medications. Detailed investigations are presented in Table 1.

**Table 1:** Laboratory parameters of the patient over four years

Laboratory parameters	April 2015	February 2016	December 2016	June 2018	August 2019	Reference ranges
White blood cell count	4.7	8.2	8.7	7.9	8.1	4.5 to 11.0 × 10^9^/liter
Hemoglobin level	7.3	9.4	8.3	8.6	8.1	13.6 to 15 grams per deciliter
Platelet count	545	458	410	297	275	150 to 400 × 10^9^/liter
Serum creatinine	40	68	30	51	61	53-106 micromoles/liter
Sodium	126	131	141	135	137	135 to 145 milliequivalents per liter (mEq/L)
Potassium	3.6	2.9	2.2	2.3	3.0	3.6 to 5.2 milliequivalents per liter (mEq/L)
Phosphorus	0.79	0.85	0.66	1.03	1.27	0.97-1.45 millimoles per liter (mmol/L)
Calcium	1.7	2.22	1.59	1.8	1.9	2.2 to 2.6 millimoles per liter (mmol/L)
Alkaline phosphatase	1000	637	465	796	490	30 to 130 units per liter
25-hydroxyvitamin D (25(OH)D)	10	22	21	12	24	Insufficiency <75 nanograms per milliliter (ng per mL) deficiency <30 nanograms per milliliter (ng per mL)
Thyroid-stimulating hormone (TSH)	4.6	4.2	3.3	3.2	1.6	0.45 and 4.5 milliunits per liter (mU/L)
Parathyroid hormone	1.67			19.6	21.1	2 to 6.8 picograms per milliliter (pg/mL)
HIV viral load	2,566,506	Undetectable	4,282,432	2,473,928	1,840	Copies per ml
CD4	12	68	85	83	169	cells/mm3

Accordingly, the diagnosis of TDF-related Fanconi syndrome was made based on serum bone minerals (Table 1) and urine parameters as shown in Table 2.

**Table 2:** Urine parameters for reported patient mEq/L: milliequivalents per liter; mmol/L: millimoles per liter; mg/dL: milligrams per deciliter

Urine parameters	Value	Reference ranges
Urine sodium	59	40-220 mEq/day
Urine potassium	32.6	25 to 125 mEq per day
Urine glucose	2.3	0 to 0.8 mmol/L
Urine phosphorus	7.6	13-28 mEq/L
Urine creatinine	23.8	40-120 mg/dL
Tubular reabsorption of phosphate	66.3%	82-95 %

She was treated for her pathological fracture conservatively with active vitamin D replacement along with calcium. The patient was also treated with combined estrogen/progesterone replacement, as she had been amenorrheic for seven months. This hormonal therapy was planned to be given until the patient reached her normal age of menopause.

At the follow-up visit three months later, the patient reported remarkable improvement in her muscle and back pain. Her gait was steadier, and her muscle strength was better. She continued on vitamin D, calcium, and estrogen/progesterone combinations, with no further fractures reported during her follow-up period. Unfortunately, the patient passed away in July 2020 due to severe COVID-19 pneumonia.

## Discussion

Tenofovir disoproxil fumarate may cause a multifactorial unfavorable effect on bone health. It may cause a Fanconi-like syndrome, negatively affect vitamin D metabolism, and/or disturb the bone mineral remodeling cycle, causing secondary hyperparathyroidism [18]. As a result, it may increase the risk of developing osteoporotic fractures with a hazard ratio of 1.04 to 1.16, accounting for the rate of one to five osteoporotic fractures per 1,000 person-years, according to a large published study [2].

Baseline renal impairment, old age, low BMI, and cumulative exposure to TDF remain the most important risk factors for the development of Fanconi-like syndrome in patients receiving TDF [2,11]. Other factors may play a role in the pathogenesis of TDF tubulopathy, such as concomitant comorbidities, co-infections, and boosted antiretrovirals. However, their association is not consistent with published data [19]. Nevertheless, as TDF toxicity is largely unpredictable, clinicians should monitor patients' renal, bone, and urine profiles at least biannually for early identification of abnormalities in attempts to prevent disease progression [12].

Data are heterogeneous on whether TDF-associated tubulopathy will recover soon after TDF discontinuation or might lead to chronic renal impairment and phosphate-wasting nephropathy [10]. In a prospective case-control study on 14 HIV-infected patients, half of the cases of TDF-related tubular toxicity (seven out of 14) recovered within the first year following drug cessation [11]. In another cohort study performed on 80 patients with HIV, about 87% showed recovery following TDF discontinuation [6]. Conversely, a clinicopathologic retrospective study of 59 consecutive renal biopsies on HIV-infected patients receiving TDF revealed incomplete resolution of TDF-related tubular toxicity or progression to chronic kidney disease in up to 50% of patients [16].

Similarly, in a cohort of 13 patients with extended follow-up, complete recovery ensued but was delayed to less than four years in most of them, and only three patients required more than five years to return to their baseline tubular function [17]. In our case, although the patient did not progress to chronic renal impairment, she suffered from the rest of the skeletal and metabolic adverse effects of TDF approximately four years after TDF discontinuation, likely representing a delayed irreversible Fanconi-related effect on the patient’s BMD.

Tenofovir disoproxil fumarate-associated effects on BMD could start as early as the first year following TDF exposure, especially in female patients with baseline osteopenia and vitamin D deficiency, as happened with our patient. Densitometry changes at the lumbar spine, even with a minimal deviation of -1 Z-score, might be an early predictor of progressive consequences for bone health in patients treated with TDF [14.13.19]. Furthermore, TDF-related pathological fractures can occur sometimes in the absence of remarkable changes in bone densitometry or even with normal BMD. Therefore, some experts suggest a spinal X-ray annually after TDF initiation to detect early bone abnormalities [13]. Of note, a meta-analysis of seven studies that included more than 700 participants reported statistically significant improvement in BMD with a mean difference of 0.4 points when supplemental vitamin D and calcium were prescribed to patients on TDF as an additional strategy to minimize the negative impact of TDF on BMD [15].

The initiation of most ART regimens might result in a decline in BMD score; multiple studies have suggested that the effect on BMD tends to be lower with TAF compared to TDF [2, 3]. Other studies on treatment experience show patients also favor switching to TAF, which carries a lower risk of BMD and might significantly improve BMD scores over two to four years [4-6]. Although some studies suggested that protease inhibitors (especially if combined with boosting agents such as cobicistat or ritonavir) might augment TDF-associated BMD loss, their findings are inconsistent and their importance is uncertain, and more recent studies considered TDF as the most important independent risk for bone loss [16, 19].

In real-world practice, the simplified HAART regimen prescription has been shifted over the last few years from TDF/protease inhibitor to TAF/integrase inhibitor-based regimens, respectively [6]. The Infectious Diseases Society of America (IDSA) endorsed a guideline on antiretroviral initiation and an expert consensus recommendation for the management of bone diseases in people with HIV. Both suggested avoidance of TDF or switching to abacavir, lamivudine, or an NRTI-sparing regimen, as well as favoring integrase-inhibitors over protease-inhibitors-based ART regimens in people at risk for osteoporosis, established osteoporosis, or in patients with renal diseases, coupled with close monitoring of BMD, optimization of calcium and vitamin D levels, and lifestyle modification [2,19].

## Conclusions

Our report showed that the TDF-associated negative impact on renal tubules and bone health can be prolonged and irreversible despite drug cessation. Early changes in renal, urine, or serum bone profiles could predict such progression early in the course. Suggested strategies to minimize TDF sequelae include a biannual renal profile and urine electrolytes. Mineral parameters, annual spinal X-rays after initiation of TDF, regular bone densitometry starting two years following drug initiation, and avoidance of TDF-containing regimens when feasible are effective TDF-sparing HAART regimens that are widely available.
